# Glucose and cholesterol stabilization in patients with type 2 diabetes mellitus with depressive and anxiety symptoms by problem-solving therapy in primary care centers in Mexico City

**DOI:** 10.1017/S1463423617000512

**Published:** 2017-09-05

**Authors:** Valerio Villamil-Salcedo, Blanca E. Vargas-Terrez, Jorge Caraveo-Anduaga, Jorge González-Olvera, Adriana Díaz-Anzaldúa, José Cortés-Sotres, Magdalena Pérez-Ávila

**Affiliations:** 1 Investigator from Department of Clinical Investigations Branch, National Institute of Psychiatry Ramón de la Fuente Muñiz, Mexico City, Mexico; 2 Chief of Community Psychiatry Service from the Clinical Services Branch, National Institute of Psychiatry Ramón de la Fuente Muñiz, Mexico City, Mexico; 3 Investigator from the Epidemiology and Psychosocial Investigations Branch, National Institute of Psychiatry Ramón de la Fuente Muñiz, Mexico City, Mexico; 4 Head of the Clinical Investigations Branch, National Institute of Psychiatry Ramón de la Fuente Muñiz, Mexico City, Mexico; 5 Investigator from Department of Genetics, Clinical Investigations Branch, National Institute of Psychiatry Ramón de la Fuente Muñiz, Mexico City, Mexico; 6 Engineer, Department of Education, National Institute of Psychiatry Ramón de la Fuente Muñiz, Mexico City, Mexico; 7 M.D. Physician from Department of Clinical Investigations Branch, National Institute of Psychiatry Ramón de la Fuente Muñiz, Mexico City, Mexico

**Keywords:** cholesterol, depression, diabetes mellitus, glucose metabolism disorders, primary healthcare

## Abstract

**Aim:**

The aim of this study was to determine if the problem-solving therapy (PST) helps control metabolic variables in patients with type 2 diabetes mellitus (T2DM) who show depressive and anxiety symptoms.

**Background:**

T2DM is a chronic-degenerative multifactorial disease. It is considered one of the main public health problems in the world, and it represents an important social and economic burden. It is frequently associated with major depression and anxiety disorders, which are related with high glycated hemoglobin (HbA_1c_) concentrations and poor metabolic control.

**Method:**

We initially included 123 patients diagnosed with T2DM from five primary care centers (PCC) in Mexico City. HbA_1c_, central glucose, and lipid profile were measured in each patient. In addition, the Kessler psychological distress scale (K-10), the Beck Depression Inventory, and the Beck Anxiety Inventory were applied at the beginning and, to those who continued, at the end of the PST, as well as four months later.

**Findings:**

In total, 36 patients completed the PST and the follow-up. There was a significant decrease in depressive and anxiety symptoms (*P*<0.001), as well as in total cholesterol (*P*=0.002), HbA_1c_ (*P*=0.05), and low-density lipoprotein (LDL) (*P*=0.022). The PST helps reduce depressive and anxiety symptoms and may help stabilize glucose and cholesterol up to four months. Further studies on this area are recommended. If our findings are confirmed, the PST could help improve the quality of life of thousands of individuals with psychiatric-metabolic co-morbidity who only visit PCC.

## Background

Type 2 diabetes mellitus (T2DM) non-insulin-dependent is a chronic-degenerative multifactorial disease. It is considered one of the main public health problems in the world, as it represents an important social and economic burden, and one of the leading causes of disability and premature death (Aguilar-Salinas *et al*., 2003; World Health Organization, 2016). In 2014, the world average health expenditure attributable to diabetes per person ranged from USD 1583 to 2842, and the annual global health expenditure ranged from USD 612 to 1099 billion (da Rocha *et al*., 2016). The International Diabetes Federation (IDF) estimates that in 2030 there will be 552 million individuals with diabetes worldwide, which implies an increase of 50.7% from 2011 to 2030, and an annual increase of 1.7 times in the adult population (Whiting *et al*., 2011). By 2035, a rise to 592 million individuals with diabetes is expected according to the IDF (Guariguata *et al*., 2014). In Mexico, The National Health and Nutrition Survey (2000, 2006, 2012, and 2016; ENSANUT, Spanish acronym) showed an increase during those years, of 5.7, 7.0, 9.2, and 9.4%, respectively (Secretaría de Salud, 2016).

Diabetes was associated with 8.4% of all-cause deaths in adults aged 20–79 years, almost 5.1 million deaths in 2013 (International Diabetes Federation & Diabetes Atlas Group, 2015). According to National Health and Nutrition Examination Survey (NHANES 1999-2000) in United States, 63% of non-institutionalized adults aged 20 years or older with previous diabetes reported a poor glycemic control [glycated hemoglobin (HbA_1c_)>7%), which is a risk factor for vascular disease (the report included estimations for different racial and ethnic groups, including Mexican American samples) (Harris *et al*., 1999; Saydah *et al*., 2004). In México, ENSANUT 2012 reported poor glycemic control in 74.4% of adults aged 20 years or older with previous diabetes (Flores-Hernández *et al*., 2015). In two districts of Mexico city poor glycemic control (HbA_1c_>6.5%) was confirmed in individuals aged 35 years or older with previous diabetes (male=79% and female=82%) (Alegre-Díaz *et al*., 2016).

Depression and anxiety disorders belong to other group of multifactorial conditions in which neurotransmission and the immune response may be affected. Both entities are highly associated with metabolic diseases involving the pro-inflammatory profile (Felger & Lotrich, 2013; Hernandez *et al*., 2013), which appears to be related to the co-morbidity between T2DM and depression/anxiety (Lustman *et al*., 1992), the latter being associated with high levels of HbA_1c_ and poor metabolic control (Lustman *et al*., 2000). It has been reported in some studies that this co-morbidity varies from 47 to 63% (Colunga-Rodríguez *et al*., 2008; Rivas-Acuña *et al*., 2011) depending on sex (Arroyo *et al*., 2004).

A pharmacotherapeutic approach has traditionally been used to treat both entities (chronic-degenerative diseases and mental disorders). However, current treatments also require psychosocial interventions that have been shown to be effective. The World Health Organization recommends the problem-solving therapy (PST) for depression disorders treatment and to reduce depressive symptomatology (World Health Organization, 2004; 2010), which is a brief intervention acting ‘in the here and now’, that helps identify a relationship between physical and emotional symptoms. The psychotherapy consists in empowerment of the patient to make changes in emotional symptoms to reduce physical symptoms. It has seven steps that are followed in 6–8 weekly sessions: Step 1 – explaining the therapy, identification, and its link to symptoms; Step 2 – clarification and understanding of the problem; Step 3 – establishment of feasible and achievable goals; Step 4 – generating solutions; Step 5 – choosing the preferred solution; Step 6 – implementation of the preferred solution; and Step 7 – evaluation (Vargas-Terrez & Villamil-Salcedo, 2013). Previous studies have shown the efficacy of the PST in reducing depressive and anxiety symptoms (Mynors-Wallis *et al*., 1995; Hassink-Franke, 2013).

Approaches for mental health services in primary care centers (PCC) have also been evaluated; the Collaborative Care is a model that has proven to be effective for chronic-degenerative diseases; it involves both training and consultation-liaison by a team of quasi-specialists (also called managers) who work with the patient and advice of General Practitioners (GPs) in PCC in order to improve the quality of healthcare (Bower & Gilbody, 2005). In Mexico, this model has been tested in PCC, with GPs (Vargas-Terrez *et al*., 2012; Villamil-Salcedo *et al*., 2017) and students of Medicine on their last year of school.

The aim of this study was to assess whether the PST applied by students of Medicine helps to improve the metabolic variables in patients with T2DM who have depressive and anxiety symptoms.

## Method

This quasi-experimental and longitudinal study was initiated in March 2014. We report the first stage of the study, which ended on January 2015. Outpatients diagnosed with T2DM were recruited from the clinic for chronic diseases (CCD) at five PCC in Mexico City. Informed consent was obtained. Enrollment was consecutive, according to their scheduled appointment at the PCC. Recruitment and enrollment was done by students of Medicine on their last year of Medical School who worked during one year in the PCC. This research was approved by the Ethics and Scientific Committees of an institute specialized in psychiatry, located in Mexico City.

## Procedure

Once patients arrived to the CCD at the PCC, their clinical mental health history was completed and the Kessler psychological distress scale (K-10) was applied as a screening tool to determine if they were probable cases or not (see Instruments section). Those who scored ⩾21 on the K-10 were invited to participate in the PST (probable cases). In total, 123 patients met inclusion criteria, 36 decided to participate and completed the study, 34 accepted but dropped out during the study, and 53 decided not to participate in the PST but they accepted completing the basal clinimetric instruments and metabolic measurements.

The Beck Anxiety Inventory (BAI) and Beck Depression Inventory (BDI) were used for those with K-10 score of 21 or higher. Patients that participated in the PST received it weekly, for 6–8 weeks. At the end of the therapy, and at follow-up four months after the completion of the therapy, the same clinimetric instruments were administered.

Metabolic measurements are taken as part of the CCD common procedures: HbA_1c_, central glucose, total cholesterol, low-density lipoprotein (LDL) cholesterol, high-density lipoprotein (HDL) cholesterol, and triglycerides. They were taken at the same stages of the clinimetric instruments (at the beginning, at the end, and four months later). Subjects with K-10⩽20 points (Vargas-Terrez *et al*., 2011) and/or with alterations of fluid and electrolyte balance were excluded from the study. In addition, individuals with suicide ideation or attempts were also excluded and referred to a psychiatric hospital. Patients who refused to participate in the study continued their usual treatment within the CCD. All this process was done by the students of Medicine on their last year of Medical School.

At the CCD, only periodic measurements of HbA_1c_ and total cholesterol are usually ordered. The rest of the metabolic variables such as central glucose, triglycerides, LDL-, and HDL cholesterol are only occasionally requested. When they are, the laboratory usually does not have enough reagents or patients cannot afford its payment. For this reason, some of the individuals are lacking part of the metabolic measurements (see Tables 3 and 4).

The PST was applied by students of Medicine on their last year of Medical School in PCC in Mexico City. Before the study, they received a 20-h training and they were monitored weekly by means of the Collaborative Care model throughout the study (one year) (Villamil-Salcedo *et al*., 2017). The training of students of Medicine, done by a psychiatrist, included Depression and Anxiety epidemiology, main characteristics of symptoms, diagnosis, treatment, identification of patients with suicidal ideations, and referral of patients. For PST, training was done by two specialists in the therapy; students participated in roll playing that were tape recorded; the aim was to evaluate and homogenize the application of the psychotherapy by the students of Medicine; they received continuous supervision of the therapy throughout the study and also feedback about main mental disorders during one year.

## Instruments

### The Kessler psychological distress scale (K-10) (Kessler *et al*., 2002)

It is a brief, self-administered scale consisting of 10 questions that assess the emotional state of the subject in the last 30 days; it has been used in Australia and United States (Australian Bureau of Statistics, 2001; Kessler *et al*., 2002) and it was validated in Mexico in individuals from primary care settings; the cut-off to consider an individual as a case was ⩾21 points; internal consistency was Cronbach’s *α*=0.901, for depression: sensitivity=78.7%, specificity=79.0%; for anxiety: sensitivity=72.4%, specificity=73.8% (Vargas-Terrez *et al*., 2011).

### BDI

It is a self-report inventory of 21 questions. It determines severity of depressive symptoms in the last week; its minimum score is 0 and the maximum is 63 points: 0–13=minimal; 14–19=mild; 20–28=moderate; 29–63=severe. This instrument has been used in the general population and clinical settings in United States, Spain, and Mexico (Beck *et al*., 1988; Jurado *et al*., 1998; Sanz & Vázquez, 1998; Sanz *et al*., 2003).

### BAI

The BAI is a self-report questionnaire. It consists of 21 questions with 0–4 Likert values that assess the severity of anxiety in the last week: 0–7=minimal; 8–15=mild; 16–25=moderate; 26–63=severe. The questionnaire has been used in international studies and in Mexico in both clinical and non-clinical settings (Kabacoff *et al*., 1997; Ulusoy *et al*., 1998; Leyfer *et al*., 2006; dos Reis Quintão, 2010).

### Statistical analysis

Frequencies, mean, standard deviation, and *χ* ^2^ were obtained to describe the socio-demographic variables and type of treatment of the sample. One-way ANOVA was used for baseline measurements and monitoring. The results were analyzed with SPSS version 21.

## Results

We enrolled 123 patients; 36 (29.27%) completed the study, 34 (27.64%) dropped out, and 53 (43.09%) did not participated in the PST. The percentage of women was similar in the three groups; there were more women (83.74%) than men; age showed statistical difference between groups [*F*(2,117)=3,329, *P*=0.039], mainly between the *lost to follow-up* and *refused to participate* groups; educational attainment [*χ* ^2^(6)=5.563, *P*=0.480] and marital status [*χ* ^2^(2)=0.357, *P*=0.880] did not show differences between the three groups; however, employment was significantly different between groups [*χ* ^2^(4)=12.701, *P*=0.007] (Table 1), mainly between the *unpaid labor work* group that completed the study and the *paid labor work* group that dropped out. Years after the onset of Diabetes were similar in the three groups [*F*(2,90)=0,401, *P*=0.671]; however type of treatment showed statistical differences for insulin [*χ* ^2^(2)=8.22, *P*=0.0164], metformin [*χ* ^2^(2)=16.82, *P*=0.0002], and other drugs used for diabetes co-morbidity [*χ* ^2^(2)=11.21, *P*=0.0037], but not for glibenclamide [*χ* ^2^(2)=0.17, *P*=0.918] (Table 2).

**Table 1:** Socio-demographic characteristics

	Completed the study [*n*=36 (29.27%)]			Lost to follow-up [*n*=34 (27.64%)]			Refused to participate [*n*=53 (43.09%)]			Total [*n*=123 (100%)]			
Group	*n*	M	SD	*n*	M	SD	*n*	M	SD	*n*	M	SD	*P*
Age													
Male	2	47.5	0.7	7	47.3	9.2	11	55.5	8.1	20	51.9	8.9	**0**.**039**
Female	34	53.1	8.4	27	49.7	9.4	42	53.4	8.7	103	52.3	8.9	
Total	36	52.8	8.2	34	**49.2**	9.3	53	**53.8**	8.6	123	52.2	8.8	
Educational level [*n* (%)]													
Without a scholarship	9 (25.00)			10 (29.41)			11 (20.75)			30 (24.39)			0.480
Primary	15 (41.67)			10 (29.41)			13 (24.53)			38 (30.89)			
Secondary	6 (16.67)			9 (26.47)			15 (28.30)			30 (24.39)			
High school	6 (16.67)			5 (14.71)			14 (26.42)			25 (20.33)			
Marital status [*n* (%)]													
Non-single	24 (66.67)			23 (67.65)			38 (71.70)			85 (69.11)			0.880
Single	12 (33.33)			11 (32.35)			15 (28.30)			38 (30.89)			
Employment [*n* (%)]													
Unpaid work	**25 (69.44)**			12 (35.29)			25 (47.17)			62 (50.41)			**0**.**007**
Paid work	10 (27.78)			**22 (64.71)**			23 (43.40)			55 (44.72)			
Unemployment	1 (2.78)			0 (0.00)			5 (9.43)			6 (4.88)			

*n*=Number of patients; M=mean; Primary=primary school (up to 6 years), Secondary=secondary school (7–9 years), High school (10 or more years); Unpaid work=person who does an activity but does not receive money for it (eg, domestic labor, student); Paid work=person who receives money for a work; Unemployment=person who did not work or study.Statistical significance values are in bold.

**Table 2:** Years after the onset of diabetes and type of treatment

Group	Completed the study [*n*=36 (29.27%)]			Lost to follow-up [*n*=34 (27.64%)]			Refused to participate [*n*=53 (43.09%)]			Total [*n*=123 (100%)]			
	*n*	M	SD	*n*	M	SD	*n*	M	SD	*n*	M	SD	*P*
Years after onset													
Male	2	7.5	9.2	6	4.7	2.8	9	6.9	5.3	17	6.2	4.8	0.671
Female	30	8.9	8.2	21	7.2	5.5	28	7.9	6.6	79	8.1	7.0	
Total	32	8.8	8.1	27	6.6	5.1	37	7.6	6.3	96	7.8	6.6	
Treatment [*n* (%)]													
Insulin	18 (50.0%)			17 (50.0%)			13 (24.5%)			48 (39.0%)			**0**.**016**
Glibenclamide	9 (25.0%)			9 (26.5%)			12 (22.6%)			30 (24.4%)			0.918
Metformin	35 (97.2%)			31 (91.2%)			35 (66.0%)			101 (82.1%)			**<0**.**001**
Other drugs	14 (38.9%)			10 (29.4%)			5 (9.4%)			29 (23.6%)			**0**.**003**
Total	36 (100.0%)			34 (100.0%)			53 (100.0%)			123 (100.0%)			

*n*=Number of patients; M=mean; Other drugs=bezafibrate, sitagliptin, pravastatin, telmisartan, and enalapril.Statistical significance values are in bold.

Mean and standard deviation of basal clinimetric and metabolic variables were similar in the three groups and no statistical differences were shown between them, except for triglycerides measurements: K-10 [*F*(2,120)=0.435, *P*=0.649]; BDI [*F*(2,83)=2.975, *P*=0.056]; BAI [*F*(2,81)=1.765, *P*=0.178]; HbA_1c_ [*F*(2,99)=2.235, *P*=0.112]; central glucose [*F*(2,100)=1.924, *P*=0.151]; total cholesterol [*F*(2,95)=0.185, *P*=0.831]; LDL [*F*(2,70)=0.895, *P*=0.413]; HDL [*F*(2,69)=2.400, *P*=0.098]; triglycerides [*F*(2,96)=3.561, *P*=0.032] (Table 3). The difference in triglycerides was mainly between *completed the study* and the *lost to follow-up* groups.

**Table 3:** Basal mean and SD of clinimetric and metabolic variables of patients that finished, dropped out, and did not accept problem-solving therapy

	Completed the study			Lost to follow-up			Refused to participate			Total				
Variable	*n*	M	SD	*n*	M	SD	*n*	M	SD	*n*	M	SD	*F*	*P*
K-10	36	27.11	6.19	34	28.03	4.92	53	26.89	5.88	123	27.27	5.68	0.435	0.649
BDI	36	24.22	8.29	30	27.47	11.93	20	19.80	13.20	86	24.33	11.14	2.975	0.056
BAI	36	18.72	10.60	27	20.67	16.13	21	13.95	9.97	84	18.15	12.63	1.765	0.178
HbA_1c_ (%)	35	8.23	2.42	34	8.22	2.72	33	7.12	2.27	102	7.87	2.51	2.235	0.112
Central Glucose (mg/dL)	35	163.30	67.17	34	172.08	87.53	34	139.32	55.71	103	158.28	71.96	1.924	0.151
Total cholesterol (mg/dL)	33	197.95	42.59	34	194.99	38.73	31	191.47	46.44	98	194.88	42.22	0.185	0.831
LDL (mg/dL)	24	120.24	31.93	30	107.12	32.60	19	109.01	49.43	73	111.92	37.41	0.895	0.413
HDL (mg/dL)	26	52.08	13.73	29	44.86	11.92	17	51.00	13.66	72	48.92	13.26	2.400	0.098
Triglycerides (mg/dL)	34	**166.91**	87.45	34	**240.30**	169.06	31	169.85	110.34	99	193.04	130.80	3.561	**0**.**032**

*n*=Number of patients; M=mean; K-10=Kessler psychological distress scale; BDI=Beck Depression Inventory; BAI=Beck Anxiety Inventory; HbA_1c_=glycated hemoglobin; LDL=low-density lipoprotein; HDL=high-density lipoprotein.Statistical significance values are in bold.

Patients who completed the PST and had a four-month follow-up showed a marked decrease in emotional distress K-10 [*F*(2,46)=38.375, *P*<0.001]; BDI [*F*(2,44)=37.817, *P*<0.001]; BAI [*F*(2,44)=15.394, *P*<0.001]; and total cholesterol [*F*(2,40)=7.251, *P*=0.002]. There was also a decrease in HbA_1c_ [*F*(2,44)=3.010, *P*=0.05] and LDL [*F*(2,24)=4.493, *P*=0.022]. The rest of the variables showed a decrease, but it was not statistically significant: glucose [*F*(2,44)=2.136, *P*=0.130]; triglycerides [*F*(2,40)=1.232, *P*=0.302]; HDL had no significant increase in three measurements [*F*(2,24)=0.505, *P*=0.610] (Table 4).

**Table 4:** Mean baseline, final and follow-up of clinimetric and metabolic variables of patients who completed the problem-solving therapy

		Baseline		Final		Follow-up			
Variable	*n*	M	SD	M	SD	M	SD	*F*	*P*
K-10	24	27.50	5.69	14.58	4.19	15.67	7.69	38.375	**<0**.**001**
BDI	23	24.78	8.36	7.43	5.52	10.87	9.39	37.817	**<0**.**001**
BAI	23	20.61	11.82	6.17	4.25	11.04	11.58	15.394	**<0**.**001**
HbA_1c_ (%)	23	8.02	2.33	7.26	1.33	7.45	1.86	3.010	**0**.**050**
Central glucose (mg/dL)	23	157.72	62.66	132.48	39.52	135.89	61.29	2.136	0.130
Total cholesterol (mg/dL)	21	200.57	33.89	172.38	32.95	169.19	45.81	7.251	**0**.**002**
LDL (mg/dL)	13	111.57	28.63	91.55	30.72	80.26	30.70	4.493	**0**.**022**
HDL (mg/dL)	13	52.08	11.54	52.3	12.47	50.08	13.52	0.505	0.610
Triglycerides (mg/dL)	21	158.38	85.62	133.23	48.61	144.44	62.69	1.232	0.302

*n*=Patients who had three measurements; M=mean; K-10=Kessler psychological distress scale; BDI=Beck Depression Inventory; BAI=Beck Anxiety Inventory; HbA_1c_=glycated hemoglobin; LDL=low-density lipoprotein; HDL=high-density lipoprotein.Statistical significance values are in bold.

Basal glycemic control (HbA_1c_<7%) was 30.43%, at the end of the therapy 50% and four month later (end of the follow-up) 56.25%.

## Discussion

In this study, from 123 patients, 36 finished the therapy (29.27%). Most of the original sample study participants (87 patients, 70.73%) did not accept to participate in the PST or dropped out during the study. Reasons maybe related to money. Patients with *unpaid labor work*, such as domestic labor, tended to complete the study and patients with *paid labor work* tended not to participate or to drop out. We corroborated this finding because some patients who did not participate said that they did not have time to attend weekly to the PCC because they did not have permission in their work to go to receive mental health services. Some patients who dropped out the therapy mentioned they did not have enough money to go to the PCC. That means that we must do changes in the primary healthcare for workers so that this population is also benefited from the mental healthcare in their workplace or elsewhere.

Regarding age, we found statistical differences between the group that dropped out (mean=49.2) and the group that did not accept the PST (mean=53.8), although we do not know the reason for this difference.

The drugs most commonly used for diabetes were insulin, metformin, and drugs to treat co-morbidity.

In basal measurements we found significant differences in triglycerides mainly between those who completed (mean=166.91) and those who dropped out (mean=240.30), but we do not know the reasons of these differences.

In the follow-up section of the study, we observed a statistically significant decrease in several variables including all the clinimetric measurements, HbA_1c_, total cholesterol, and LDL. As slight increase was identified for HDL, without representing a significant difference, but some other variables were not significantly different (central glucose and triglycerides). A larger number of patients may be needed to confirm this result (see Table 4). We consider an important finding that PST can help reduce or stabilize metabolic variables at four-month follow-up.

On other hand, in this study we addressed various aspects. First, whether there were direct benefits with the use of the PST in patients with T2DM; if there was an improvement in their metabolic parameters. Second, if there was a relationship between T2DM, depression, and anxiety symptoms, and if this could be measured. We were interested in knowing if the PST is useful in PCC, and if the Collaborative Care model between students of Medicine on their last year of Medical School and investigators could help improve the mental health of patients in the PCC.

Even if central glucose did not significantly decrease, as HbA_1c_ did, there was a stable change in glucose over time. In fact, glycemic control increased from 30.43% in the basal measurement to 50% at the end of the PST and to 56.25% four months later. Thus, PST had a positive impact on diabetes status as well as in lipid profile, reducing cardiovascular risk. The PST may promote self-care of patients, and it may allow them to solve their daily problems, to reduce stress, which may result in greater adherence to their medical appointments and treatment; therefore, it may help stabilize their glucose and lipid profile. However, it is necessary to do further studies to evaluate the persistence of changes over time.

Our results support the idea that the PST is useful and practical for mental healthcare in PCC, mainly to reduce depressive and anxious symptoms but also glucose and lipid stabilization. In this study, changes persisted up to a four-month follow-up. Previous studies in primary care reflect the same results and conclude the usefulness of this therapy in PCC (Mynors-Wallis *et al*., 1995; Kendrick *et al*., 2006; Hassink-Franke, 2013).

The association between T2DM and depressive and anxious symptoms is very strong; our study showed that the association is related with the tendency observed in patients, to remain decompensated for longer periods; similar findings have been reported (Knol *et al*., 2006; Moussavi *et al*., 2007; Icks *et al*., 2008; Castro-Aké *et al*., 2009). Thus, the improvement in their mental health may help improve their medical condition (Bystritsky *et al*., 2014).

Both HbA_1c_ and total cholesterol statistically decreased when depression and anxiety symptoms were dealt with. Hence, our study illustrates the following slogan promoted by the *Lancet Journal* in 2007: ‘There is no health without mental health’ (Prince *et al*., 2007).

It is important to include a psychotherapeutic support on plans and actions aimed at treating medical problems. This dual treatment will promote the general improvement of patients.

About PST, most of the studies reported that GPs, residents in Family Medicine, nurses and psychologists may apply the PST (Kendrick *et al*., 2006; Hassink-Franke, 2013; Bosmans *et al*., 2012). We did not identify studies in which students of Medicine on their last year of Medical School were PST therapists, but there is no reason to believe this could affect the results. In fact, they were very interested in participating in this research, and personal motivation may be a key factor in treatment success in primary care settings (Villamil-Salcedo *et al*., 2006). GPs of PCC did not participate in this project because their lack of interest in mental health and because they were overloaded with other activities. These same limitations were observed in other studies carried out in Mexico on mental health in the primary healthcare (Villamil-Salcedo *et al*., 2017).

In any case, an important aspect in the implementation of any therapeutic technique is the training of the personnel, the application and the supervision of the therapy; these were made with video filming and role playing, as well as weekly follow-ups of the cases through a Collaborative Care model. The results from several studies suggest that Collaborative Care allows one to build a bridge between PCC and specialists (Primary and Tertiary Healthcare), and that this collaboration improves primary healthcare (Gask *et al*., 1997; Bower & Gilbody, 2005; MacDonald *et al*., 2008; Hassink-Franke, 2013; Villamil-Salcedo *et al*., 2017).

In our project, and taking into consideration previous results, we consider that the Collaborative Care model is very useful; it is a means to maintain continuous contact with the staff at PCC, to help them address most common mental health conditions. Weekly monitoring of physicians showed a positive, short to medium-term impact.

From the results of this first stage of our study, we have initiated an experimental study with a four-month follow-up. We are including a higher number of PCC, and the study was evaluated by the ClinicalTrials.gov Protocol Registration (ID: NCT02889172) in order to have results available to other investigators. In addition, we followed the CONSORT recommendations for clinical trials to obtain results that can be extrapolated.

One of the major limitations of the studies in primary care in Mexico is that it is difficult to periodically and routinely assess all important metabolic variables such as LDL, HDL, and triglycerides. This is because the PCC do not have adequate infrastructure or inputs required for all studies; on the other hand, patients who attend these PCC belong to a low economic level, so often they cannot afford the studies.

These barriers, typical of the study population, limit long-term monitoring. They were disclosed to authorities for decision-making and modifications thereof, but changes require long-term strategies.
